# Congestive Hepatopathy Secondary to Right Ventricular Hypertrophy Related to Monocrotaline-Induced Pulmonary Arterial Hypertension

**DOI:** 10.3390/ijms222111891

**Published:** 2021-11-02

**Authors:** Douglas Mesadri Gewehr, Allan Fernando Giovanini, Beatriz Alvarez Mattar, Anelyse Pulner Agulham, Andressa de Souza Bertoldi, Seigo Nagashima, Fernando Bermudez Kubrusly, Luiz Fernando Kubrusly

**Affiliations:** 1 Department of Medicine, Mackenzie Evangelical School of Paraná, Curitiba 80730-000, Brazil; afgiovanini@gmail.com (A.F.G.); beatrizamattar@gmail.com (B.A.M.); anelyseagulham@gmail.com (A.P.A.); andressa_bertoldi@hotmail.com (A.d.S.B.); kubrusly@incorcuritiba.com.br (L.F.K.); 2 Denton Cooley Institute of Research, Science and Technology, Curitiba 80730-201, Brazil; kubrusly.f@gmail.com; 3 Curitiba Heart Institute, Curitiba 80730-201, Brazil; 4 Laboratory of Experimental Pathology of Health and Biological Sciences, Pontifical Catholic University of Paraná, Curitiba 80215-901, Brazil; seigocap@gmail.com

**Keywords:** liver disease, congestive hepatopathy, hypertrophy, right ventricular, pulmonary arterial hypertension, monocrotaline, liver fibrosis, sinusoidal dilatation

## Abstract

Heart dysfunction and liver disease often coexist. Among the types of cardiohepatic syndrome, Type 2 is characterized by the chronic impairment of cardiac function, leading to chronic liver injury, referred to as congestive hepatopathy (CH). In this study, we aimed to establish a rat model of CH secondary to right ventricular hypertrophy (RVH) related to monocrotaline (MCT)-induced pulmonary arterial hypertension (PAH). Fifty male Wistar rats were divided into four groups and randomly assigned to control and experimental groups. Three experimental groups were submitted to intraperitoneal MCT inoculation (60 mg/kg) and were under its effect for 15, 30 and 37 days. The animals were then sacrificed, obtaining cardiac and hepatic tissues for anatomopathological and morphometric analysis. At macroscopic examination, the livers in the MCT groups presented a nutmeg-like appearance. PAH produced marked RVH and dilatation in the MCT groups, characterized by a significant increase in right ventricular free wall thickness (RVFWT) and chamber area. At histological evaluation, centrilobular congestion was the earliest manifestation, with preservation of the hepatocytes. Centrilobular hemorrhagic necrosis was observed in the groups exposed to prolonged MCT. Sinusoidal dilatation was markedly increased in the MCT groups, quantified by the Sinusoidal Lumen Ratio (SLR). The Congestive Hepatic Fibrosis Score and the Centrilobular Fibrosis Ratio (CFR) were also significantly increased in the MCT_30_ group. Hepatic atrophy, steatosis, apoptotic bodies and, rarely, hydropic swelling were also observed. SLR correlated strongly with CFR and RVFWT, and CFR correlated moderately with RVFWT. Our rat model was able to cause CH, related to monocrotaline-induced PAH and RVH; it was feasible, reproducible, and safe.

## 1. Introduction

Heart failure (HF) is a clinical syndrome characterized by dyspnea and/or exertional limitation, since it involves an impairment of ventricular filling, or even the ejection of blood. It is noteworthy that this comorbidity has become a major public health problem in recent decades, not only due to its increasing prevalence and the advance of medical therapies, but also due to its association with other pathological entities [1].

In this context, heart dysfunction and liver disease often coexist as they are systemic disorders that share similar etiological factors in their development. Accumulating evidence indicates that organ crosstalk and interaction occur between the heart and liver in a manner that is comparable to what is known for cardiorenal syndrome [2]. Based on this analogy, a cardiohepatic syndrome (CHS) in HF was proposed by Poelzl and Auer [2], classifying this syndrome into five types.

Among the types of cardiohepatic syndrome, Type 2 CHS is characterized by the chronic impairment of cardiac function, leading to chronic liver injury, which is commonly referred to as congestive hepatopathy [3,4]. The incidence of congestive hepatopathy is 15% to 65% in severe HF [3]. The primary pathophysiologic mechanism in liver dysfunction is elevated right-sided cardiac pressure transmitted caudad to the hepatic and portal venous circulation, with elevated central venous pressure and normal hepatic venous pressure gradient, leading to passive hepatic congestion. Other important mechanisms includes decreased hepatic blood flow and decreased arterial oxygen saturation (more related to type 1 CHS) [2,3,5].

Generally, congested liver is characterized as a “nutmeg liver”, with dark centrilobular zones, reflecting sinusoidal congestion, alternating with pale periportal zones. The most common histologic features includes sinusoidal dilatation, congestion and hepatocyte atrophy, most prominent in zone 3. Chronic congestion precipitates the extravasation of fluid and erythrocytes into the space of Disse, leading to inflammation, hemorrhagic necrosis, centrilobular and periportal fibrosis and, eventually, cirrhosis [5,6,7]. 

The interactions between the liver and heart have been the subject of significant interest and investigation, requiring a well-characterized animal model, since their pathophysiology is not completely understood [8]. Monocrotaline (MCT) is a pyrrolizidine alkaloid plant derived from the seeds of the Crotalaria spectabilis. The MCT alkaloid is activated to the reactive metabolite in the liver by P-450 cytochrome. After metabolic activation, MCT causes a variety of toxic insults that vary by dosage and include pulmonary endothelial injury, an increase in vasoconstrictors and thickening of the vascular wall (mainly smooth muscle cells), leading to an increase in pulmonary resistance, pulmonary hypertension and right heart failure [9,10]. 

Although there are several studies that describe some histological findings of passive hepatic congestion secondary to MCT-induced right heart dysfunction, none of them performed graduation or characterized the anatomopathological evolution of congestive liver disease in a rat model.

The aim of this study was to establish a rat model of congestive hepatopathy secondary to right ventricular hypertrophy related to monocrotaline-induced pulmonary arterial hypertension by quantifying relevant histological findings, hepatic fibrosis, sinusoidal dilatation and the longitudinal progression of the disease. This is the first experimental study to evaluate the relationship between sinusoidal dilatation, hepatic fibrosis and cardiac morphometric parameters in a right ventricular hypertrophy rat model. 

## 2. Results

### 2.1. Effects of Monocrotaline on Physical Characteristics and Survival

**Figure 1:**
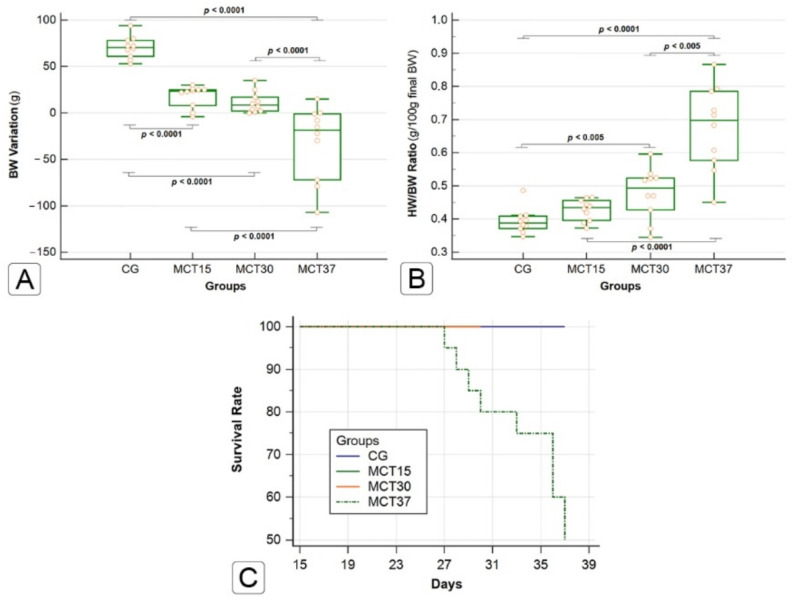
Body weight (BW) variation (**A**) and HW/BW ratio (**B**); g/100 g final BW, are expressed as median and interquartile range (Kruskal–Wallis method followed by Simes–Hochberg method). Mortality was observed daily, and the figure shows the survival rate at each time of the different experimental groups, through a Kaplan–Meier curve (**C**).

The MCT-injected animals exhibited signs of cardiorespiratory disease, such as decreased body weight, anorexia, weakness, variable degrees of dyspnea, intolerance to efforts and cyanosis of the extremities. These signs became more evident in the MCT_30_ and MCT_37_ groups. The effects of MCT on body weight (BW) and heart weight/body weight ratio (HW/BW ratio) are shown in Figure 1A,B. A decrease in BW gain was observed as the animals were exposed to the effects of MCT for a longer time, so that the animals of MCT_37_ experienced a significant reduction, of about 11%, in their final BW in comparison to their initial BW (CG vs. MCT_30_ and MCT_30_ vs. MCT_37_; *p* ≤ 0.0001). The CG showed a constant BW gain during the study period. The HW/BW ratio, g/100 g final BW, was significantly increased in the MCT_30_ and MCT_37_ groups compared to the CG (*p* < 0.005). All the animals in the CG, MCT_15_ and MCT_30_ groups survived for the entire experimental period. Approximately one month after MCT injection, deaths began to be observed in the MCT_37_ group, with a 50% survival rate (Figure 1C).

### 2.2. Macroscopic Findings

**Figure 2:**
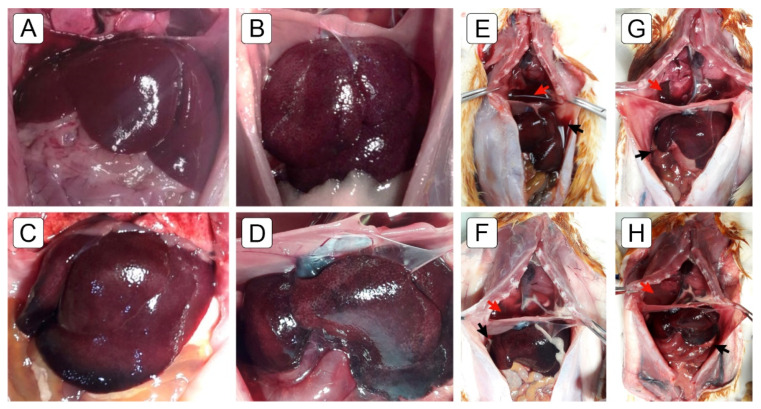
Gross macroscopic findings in (**A**) the normal liver of a control animal and (**B**–**D**) the livers of the MCT-group animals. (**B**,**C**) The livers of the MCT_30_ and MCT_37_ groups with nutmeg appearance and darkish color. In (**D**) a liver of an animal in the MCT_ED_ group with a nutmeg-like appearance, apparent reduction in size and volume and darkish color occupying a larger area of the organ’s surface. The MCT_37_- and MCT_ED_-group animals showed bloody ascites (black arrows) and pericardial and pleural (red arrows) effusion (**E**–**H**).

At sacrifice, macroscopic examination of the livers in the MCT group showed surface congestion with a darkish color, suggesting nutmeg-like liver congestion (Figure 2B–D). These changes were not observed in the CG (Figure 2A). Differences in liver size and weight was not observed between the groups. The accumulation of bloody ascites and pericardial and pleural effusion were also observed (Figure 2E–H). These changes were not observed in the CG. The animals in the MCT_ED_ group presented a macroscopic aspect similar to the MCT groups during the postmortem examination; however, they also presented an apparent reduction in size-volume, with blackish coloration occupying a larger area of the organ’s surface (Figure 2D).

**Figure 3:**
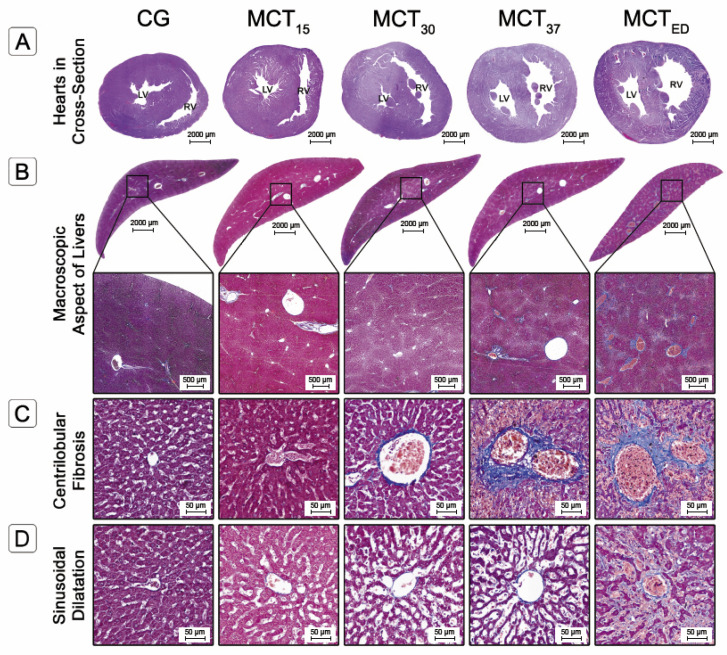
(**A**). Heart cross-section stained with hematoxylin-eosin of a CG animal and MCT-group animals (Hematoxylin-eosin stain). (**B**) Macroscopic aspect of livers stained with Masson trichrome. (**C**,**D**) Regions of interest (ROIs) of liver tissue sections stained with Masson trichrome of the CG animal and MCT-group animals, showing centrilobular fibrosis (**C**) and sinusoidal dilatation (**D**). LV, left ventricle; RV, right ventricle.

**Figure 4:**
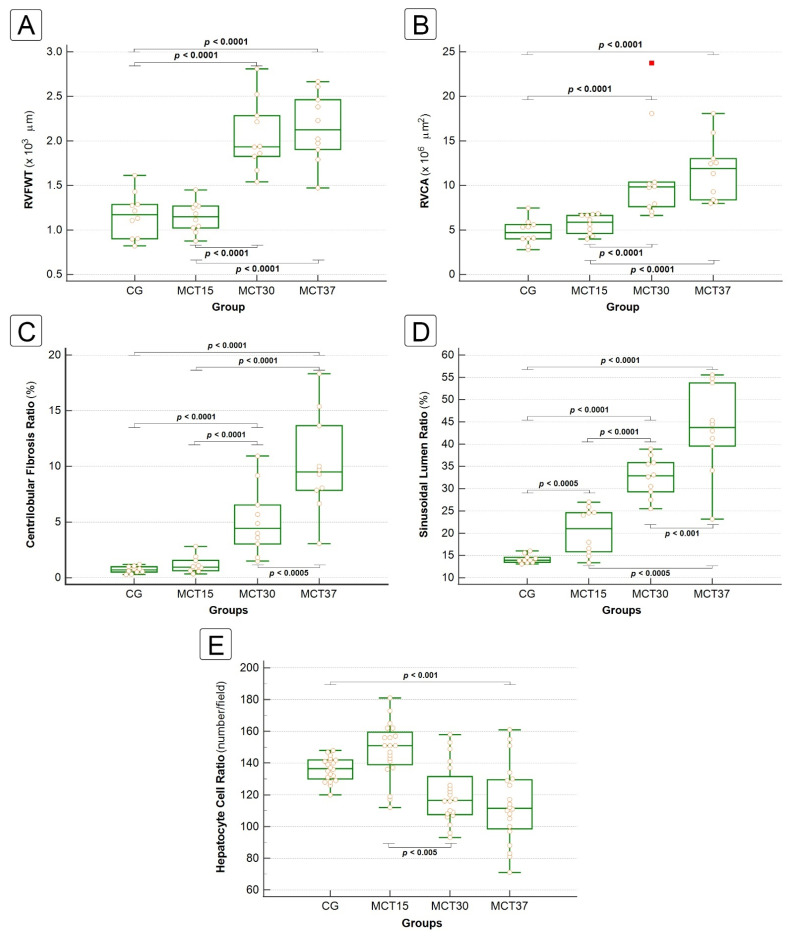
Cardiac and hepatic morphometric analysis. The box-plot graphs (median and interquartile range) show right ventricular free wall thickness (RVFWT) (**A**), right ventricle chamber area (RVCA) (**B**), centrilobular fibrosis ratio (**C**), sinusoidal lumen ratio (**D**) and hepatocyte cell ratio (**E**). (**A**–**D**) *p*-values were obtained using the Kruskal–Wallis method followed by the Simes–Hochberg method. (**E**) *p*-values were obtained by ANOVA one-way with Tukey’s post-hoc test.

MCT-induced pulmonary arterial hypertension produced marked right ventricular hypertrophy and dilatation (Figure 3A), characterized by an increase in right ventricular free wall thickness (RVFWT) (Figure 4A) and chamber area (RVCA) (Figure 4B) in the MCT_30_ and MCT_37_ groups compared to the CG (*p* < 0.0001). 

### 2.3. Histological Evaluation

A total of one hundred digitalized slides were subject to detailed histological evaluation. The qualitative parameters were scored according to the criteria described in the Materials and Methods sections, and the findings are summarized in Table 1. The morphometric parameters of the heart and liver are presented in Figure 4. No findings consistent with liver abnormalities were found in the CG.

#### 2.3.1. Liver Fibrosis

The CHFSs and Centrilobular Fibrosis Ratios (CFR) were significantly increased in the MCT_30_, MCT_37_ and MCTED groups compared with the CG (0.71 ± 0.33, 5.11 ± 3.19, 10.2 ± 5.22 and 14.65 ± 7.16%, respectively; *p* < 0.0001) (Figure 4C). In the CG, the CHFSs were all score 0; in the MCT_15_ group, the score was predominantly 0; in the MCT_30_ and MCT_37_ groups, the score was predominantly 1, that is, central zone and perisinusoidal fibrosis; while in the MCT_ED_ group, the CHFSs were all ≥ 1, with a predominance of score 2, that is, centrilobular and portal fibrosis (Table 1). The CHFS was highly correlated with the centrilobular fibrosis ratio. The evolution of the liver fibrosis pattern is shown in Figure 3C.

#### 2.3.2. Sinusoidal Dilatation and Hepatocyte Cell Count

Histological examination revealed that sinusoidal dilatation was markedly increased in the MCT-group animal livers (Figure 3D), which was associated with centrilobular hepatic cord atrophy (Figure 4A). Furthermore, the sinusoidal lumen ratio (SLR), assessed by a meticulous morphometric analysis, was significantly increased in all the MCT-group animal livers (14.22 ± 1.14, 20.47 ± 5.14, 32.65 ± 4.90, 43.50 ± 12.59 and 59.38 ± 17.22%, respectively; *p* < 0.0001) (Figure 4D), with an SLR in the MCT_37_ group 3× higher than that of the CG.

The Hepatocyte Cell Ratio (HCR) was significantly decreased in the MCT_37_ and MCT_ED_ groups compared to the CG (136.1 ± 7.61, 114.15 ± 24.60 and 69.1 ± 23.33 number hepatocyte per field, respectively; *p* < 0.001) (Figure 4E). These results showed that there was significantly increased hepatocyte cell death from the 30th day.

#### 2.3.3. Congestion and Centrilobular Hemorrhagic Necrosis

**Figure 5:**
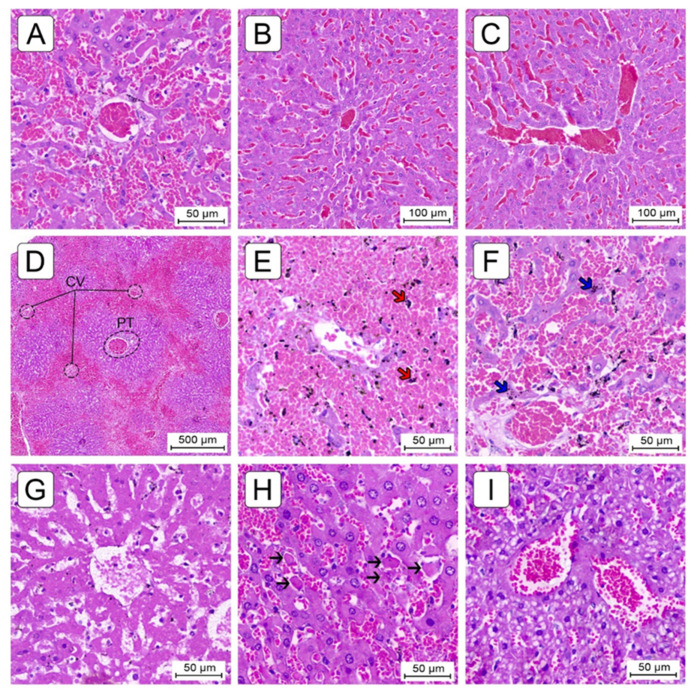
Histological changes in the livers of MCT animals. (**A**) Sinusoidal dilatation and congestion in centrilobular zone associated with the atrophy of centrilobular hepatocytes, with marked thinning of liver cell plates. (**B**,**C**) Sinusoidal congestion in zones 3 and 2 with preservation of centrilobular hepatocytes and liver cell plate thickness. (**D**) Marked centrilobular necrosis with bridging from one centrilobular vein (CV) to another. Intervening portal tracts (PT) and periportal parenchyma (zone 3) are preserved. (**E**,**F**) Centrilobular hemosiderin deposition, with the presence of dark brown granules inside the hepatocytes (blue arrows) and Kupffer cells (red arrows). (**G**) Coagulative necrosis of hepatocytes surrounding the CV, showing dense acidophilic cytoplasm associated with nuclear pyknosis, loss of nuclear details or absence of nuclei. (**H**) Hepatocyte apoptosis in early stage. Apoptotic bodies (black arrows) are generally surrounded by a clear halo and feature a condensed and eosinophilic cytoplasm, in contrast to adjacent normal hepatocytes, and either are devoid of chromatin or contain pyknotic or fragmented nuclear material. (**I**) Microvesicular steatosis, characterized by variably enlarged hepatocytes with very fine fat vacuoles. Hematoxylin-eosin stain.

Centrilobular sinusoidal congestion (SC) was the earliest manifestation and consisted of the engorgement of the centrilobular sinusoids by red blood cells (RBC’s), with the preservation of hepatocytes. Mild SC was already present in the MCT_15_ group (Figure 5B,C), significantly increased when compared to CG (*p* < 0.05), and progressed to moderate in the MCT_30_ group and severe in the MCT_37_ group (Table 1). Sinusoidal dilatation was also associated with centrilobular congestion in several cases (Figure 5A).

Centrilobular hemorrhagic necrosis (CHN) was a later manifestation and consisted of centrilobular congestion associated with frank necrosis of zone 3 hepatocytes. Moderate-severe CHN presented only in the MCT_30_ and MCT_ED_ groups and was significantly increased when compared to the CG (*p* < 0.05) (Table 1). In some animal livers, the hemorrhagic necrosis and sinusoidal congestion were too marked and severe, forming a bridging pattern between central veins (Figure 5D). Subendothelial hemorrhage was a common manifestation associated with SC and CHN, rated as mild in the MCT_30_ group and moderate-severe in the MCT_37_ and MCT_ED_ groups, significantly increased when compared to the CG (*p* < 0.05) (Table 1). 

In most cases in the MCT_37_ and MCT_ED_ groups, centrilobular hemosiderin deposition was observed, with the presence of dark brown granules inside the hepatocytes and Kupffer cells (Figure 5E,F). In rare cases, the centrilobular necrosis of hepatocytes was coagulative (Figure 5G), affecting the focal regions of the hepatic parenchyma, with these cells showing dense acidophilic cytoplasm associated with nuclear pyknosis, loss of nuclear details or absence of nuclei.

#### 2.3.4. Miscellaneous Lesions

Other histologic lesions of little or uncertain clinical significance were encountered in the animal livers. These included steatosis (Table 1), apoptotic bodies (Figure 5H) and, rarely, hydropic swelling. The steatosis was almost exclusively microvesicular (Figure 5I) and appeared first in the MCT_30_ group and rated as mild; it was maintained in the MCT_37_ group and progressed to moderate in the MCT_ED_ group.

### 2.4. Correlation between Hepatic and Cardiac Morphometric Parameters

**Figure 6:**
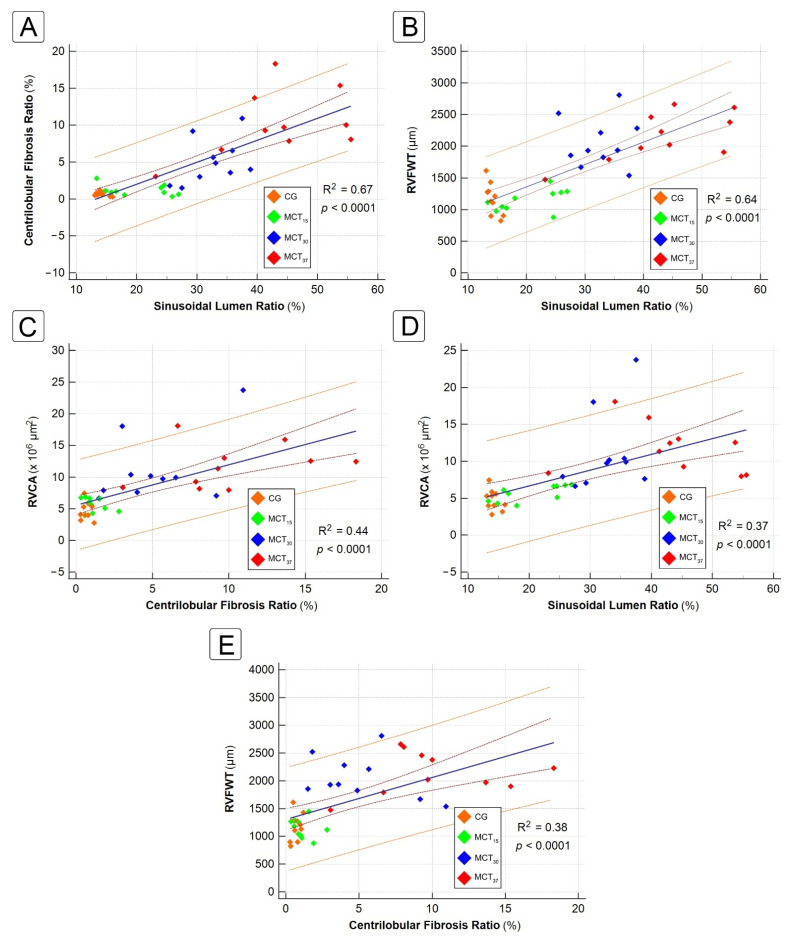
Linear regression analysis of the relationships between centrilobular fibrosis ratio, sinusoidal lumen ratio and cardiac morphometric parameters. (**A**) Correlation between centrilobular fibrosis ratio and sinusoidal lumen ratio. (**B**) Correlation between sinusoidal lumen ratio and RVFWT. (**C**) Correlation between centrilobular fibrosis ratio and RVCA. (**D**) Correlation between sinusoidal lumen ratio and RVCA. (**E**) Correlation between centrilobular fibrosis ratio and RVFWT. RVFWT, right ventricle free wall thickness; RVCA, right ventricle chamber area.

The relationship between centrilobular fibrosis, sinusoidal dilatation and cardiac parameters is shown in Figure 6. There was a strong positive correlation between CFR and SLR (R^2^ = 0.67, *p* < 0.0001) and between SLR and RVFWT (R^2^ = 0.64, *p* < 0.0001). There was a moderate positive correlation between RVCA and hepatic parameters [CFR (R^2^ = 0.44, *p* < 0.0001) and SLR (R^2^ = 0.38, *p* < 0.0001)]. Finally, there was a moderate positive correlation between RVFWT and CFR (R^2^ = 0.37, *p* < 0.0001).

## 3. Discussion

The MCT-induced PH model has been used for over 50 years, and it has been the most commonly used model in experimental studies of pathophysiology and pharmacology [10]. With it, progress has been made in understanding of the disease and a large number of the medications currently used were tested in preclinical studies with this model [11]. The present study is the first to describe in detail the histopathology of the liver in an experimental model of congestive hepatopathy (CH) secondary to monocrotaline-induced right ventricular hypertrophy and pulmonary hypertension.

The liver is a highly vascular organ that receives approximately 25% of cardiac output. Receiving blood from two different vessels, the portal vein and the hepatic artery, the liver is protected from damage by hypoflux and hypoxia. On entering the liver, blood from the portal vein and hepatic artery mix and flow through the sinusoids, in contact with hepatocytes, and drains to the centrilobular veins. The hepatic vein, formed by the confluence of the centrilobular veins, carries out the blood to the inferior vena cava, which carries blood to the right atrium [12,13]. 

It is known that any obstruction to hepatic venous outflow can result in a spectrum of clinical abnormalities, ranging from acute hepatic failure to passive hepatic congestion, depending on the acuity and level of obstruction [14]. Hepatic venous outflow obstruction (HVOO) can be divided into three categories, according to Bayraktar et al. [14]: veno-occlusive disease (VOD), at the level of the sinusoids and terminal venules; Budd-Chiari syndrome (BCS), from the hepatic veins to the superior end of the inferior vena cava; and venous obstruction at the level of the heart, referred to as CH, the focus of the present study. The etiology and clinical presentation of these abnormalities are entirely different; however, the histological findings in all three syndromes are almost identical and include sinusoidal congestion and centrilobular necrosis, which eventually leads to bridging fibrosis between adjacent central veins [15,16]. 

The disturbance of the physiological crosstalk between the heart and the liver can lead to what is known as cardiohepatic syndrome (CHS). This condition embodies the bidirectional nature of heart–liver interactions and is characterized by a vast array of interrelated derangements [2,3,17]. According to Poelzl and Auer [2], CHS is classified into five types. Type 2 CHS is characterized by the chronic impairment of cardiac function, leading to chronic liver injury, which is commonly referred to as cardiac or congestive hepatopathy. 

Congestive hepatopathy is related to chronic cardiopathy in about 90% of cases, which is known as type 2 CHS [18,19]; its incidence is estimated to be between 15% to 65%. In a recent series, the major cause of CH was ischemia heart disease, representing about 50 to 70% of cases, followed by valvular cardiomyopathy, which only accounted for 18% [18,20]. In a series from 1961, 7.2% of CH were related cor pulmonale and 2.9% to congenital diseases [21]. 

The fundamental mechanisms underlying cardiac hepatopathy are reduced arterial perfusion, whose deleterious effects are amplified by concomitant hypoxia, and passive congestion secondary to increased systemic venous pressure. The lack of valves in the hepatic veins allows increased inferior caval pressures to hit the sinusoidal bed without any attenuation. The resulting congestion produces liver damage through several pathogenic mechanisms: (1) shear stress promotes fibrogenesis and sinusoidal ischemia through the activation of hepatic stellate cells and through a decrease in nitric oxide production from endothelial cells; (2) decreased portal and arterial inflow aggravates hepatic ischemia [22,23]. 

The typical features of liver fibrosis in patients with cardiac failure consist of a spectrum of changes that depends on the chronicity of the disease [24]. The congestive liver explant has been called “nutmeg liver”, due to the presence of dark centrilobular zones, reflecting sinusoidal congestion, alternating with pale periportal zones with normal or fatty liver tissue [25], as observed in the congested livers of the rats in our study. Characteristic histological findings include sinusoidal dilatation and congestion, hepatocyte atrophy, most prominent in zone 3, extravasation of red blood cells into the space of Disse and centrilobular necrosis. With chronic cardiac dysfunction, as the liver disease progresses, bridging fibrosis typically extends between the central veins to produce a pattern that has been called “reverse lobulation” [5,6,7,13,26,27]. 

Traditional scores were developed in past years to evaluate the pattern and severity of liver fibrosis, such as METAVIR. However, these scores may not be precise enough in the context of CH, due to the reversed lobulation pattern of fibrosis observed in CH. Dai et al. [6]. recently introduced a Congestive Hepatic Fibrosis Score (CHFS), a four-graded system for the histological scoring of liver fibrosis in patients with CH. 

Although these scores attempt to standardize the analysis of the pattern of fibrosis for a known etiologic and pathophysiologic condition, they fail to explore the influence of the various cardiohepatic morphometric variables on liver fibrosis. Therefore, studies that offer this information, whether clinical or animal, are considered important.

The correlation between hepatic fibrosis ratio and cardiac hemodynamics and morphometric parameters has been reported in previous studies [5,28,29]. The degree of sinusoidal dilatation is positively correlated with the degree of elevation of right atrial pressure and inferior vena cava pressures. In an experimental rat model, using pulmonary artery banding to induce right heart failure, Fujimoto et al. [29] found that hepatic fibrosis progressed more in rats with increases in right atrium dimension (R^2^ = 0.56). Arcidi et al. [30], in an autopsy series, reported an association between chronic passive congestion and increases in right atrial size. We found similar results in our study, which showed a strong positive correlation between SLR and RVFWT (R^2^ = 0.64), a moderate positive correlation between CFR and RVFWT (R^2^ = 0.37) and a strong positive correlation between CFR and SLR (R^2^ = 0.67).

A limitation of our study is that we did not examine the biochemical parameters of the liver, as performed in other studies [5]. The elevation of serum cholestasis markers is characteristic of CH (direct bilirubin, alkaline phosphatase and gamma-glutamyl transferase); it is dependent on the increase in the central venous pressure, pressure in the right atrium and, consequently, on hemodynamic parameters [19,31]. Despite this hemodynamic correlation and the advantage it offers to the prediction of all-cause mortality in patients with heart failure with cholestasis markers [19], our method of quantifying liver damage through a histological computerized system seems to be more reliable than blood chemical examination, according to studies performed by Fujimoto et al. [29], because it involves analysis of changes in liver biochemistry that are less obvious in animal studies, even with the development of liver fibrosis.

The major clinical implication of this experimental study of congestive liver disease is the importance of considering liver fibrosis as a differential diagnosis in patients with right heart dysfunction. The effects of right heart failure on liver function may be silent, not detected by physical examination, becoming problematic with the chronicity of the cardiac disease, particularly in patients with adult congenital heart disease. In this context, a routine blood chemistry examination for liver function, especially canalicular membrane enzymes [2,32], associated with liver elastography [33,34], seems to be the most promising non-invasive method for assessing liver damage caused by cardiovascular disease. 

## 4. Materials and Methods

### 4.1. Ethical Aspects

The study protocol was approved by the Ethics Committee on the Use of Animals, Mackenzie Evangelical College of Paraná (Faculdade Evangélica Mackenzie do Paraná–FEMPAR). All the experimental protocols were performed in compliance with the National Institutes of Health (NIH) guidelines (Bethesda, MD, USA) for the care and use of laboratory animals (NIH Publication no. 85723, revised 1996) and conformed to the previously described principles and regulations for animal experimentation of Experimental Physiology (Grundy, 2015), and all steps were taken to minimize the animals’ pain and suffering during the experiments. Institutional ethical approval code: 2577/2020.

### 4.2. Experimental Animals

Fifty male Wistar rats, species Rattus norvegicus, weighing 150–250 g and aged 10–12 weeks, were used for the study and were maintained under standard laboratory conditions (temperature 25 ± 2 °C; relative humidity 50 ± 15%; and natural dark/light cycle). They were housed no more than four to a cage on corn cob bedding. They were allowed food (normal laboratory animal diet provided through a vivarium) and water ad libitum.

### 4.3. Drugs and Chemicals

Monocrotaline (MCT; Sigma-Aldrich, St. Louis, MO, USA) was weighed and dissolved in 1.0 N HCl, which was then neutralized to pH 7.4 by adding 1.0 N NaOH and distilled water was added to make up the volume. A single injection of 60 mg/kg of monocrotaline known to stimulate PAH and right heart remodeling was administered intraperitoneally.

The chemicals used for the anesthesia were: xylazine hydrochloride 2% (Xilazin^®^; Syntec, São Paulo, Brazil) and ketamine hydrochloride 10% (Cetamin^®^; Syntec, São Paulo, Brazil).

### 4.4. Experimental Design—Induction of Pulmonary Arterial Hypertension

Initially, the animals were allocated into four groups by simple randomization: control group (CG) (*n* = 10), 15-day monocrotaline group (MCT_15_) (*n* = 10), 30-day monocrotaline group (MCT_30_) (*n* = 10) and 37-day monocrotaline group (MCT_37_) (*n* = 20). The CG animals received an intraperitoneal saline solution injection (0.9% NaCl). The MCT-group animals received at D0 one single intraperitoneal dose of monocrotaline 60 mg/kg and were observed for 15 days, 30 days and 37 days, representing the MCT_15_, MCT_30_ and MCT_37_ groups, respectively. Through simple randomization and intraperitoneal administration of the drug, possible confounding factors were significantly reduced.

After the respective experimental periods, the animals were anesthetized, based on our institute’s protocol, with a combination of 0.3 mg/kg of xylazine hydrochloride 2% and 10 mg/kg of ketamine hydrochloride 10%. They were then weighed before being sacrificed through cardiac puncture exsanguination. The liver and hearts were carefully removed, dissected and weighed, using a precision semi-analytical balance (AD200; © Marte Científica, São Paulo, Brazil). The data of the animals that died during the experimental period of the MCT37 group were not included in the original group analysis. These animals were submitted to a postmortem examination and their data were compiled in the early death (MCT_ED_) group.

Monocrotaline-induced pulmonary arterial hypertension has already been reproduced and validated by many authors. In this article, we do not focus on the pulmonary histopathological aspects of PAH, since it has already been the subject of a previously published paper by our research group [9]. 

### 4.5. Histopathology

#### 4.5.1. Histological Preparation

The livers and hearts were excised and fixed in a 10% buffered formalin solution for 48 h. The largest right and left hepatic lobe were trimmed in cross-section. The hearts were trimmed in cross-section at the middle third of the ventricles. After fixation, the tissue was embedded in paraffin blocks and, later, two 4–micrometer coronal histological sections were obtained for each animal. The histological sections were stained with Hematoxylin-Eosin (HE) and Masson Trichrome (MT) and mounted on glass slides. Two slides of liver samples (HE and MT) and one slide of heart sample (HE) were confectioned per animal, totalizing one hundred and fifty slides. 

#### 4.5.2. Digital Slide Scanner and Image Acquisition

The slides of liver and heart tissue were digitalized with a scanner (Axio Scan Z1, Zeiss, Jena, Germany) (40× magnification) and the images were analyzed using ZEN 2.3 (blue edition) software (© Carl Zeiss Microscopy GmbH, 2011), which allows the user to navigate through Carl Zeiss Image (CZI) and perform geometric and quantitative measurements.

After scanning the MT liver sections with the Axio Scanner, five histology regions of interest (ROIs) per slide (images taken at full resolution with a ROI set at 1364 × 1364 pixels, with a pixel size of 4.55 µm × 4.55 µm), with the centrilobular vein in the center of each ROI, were obtained randomly from across the entire digital slide, as described above, totalizing 250 ROIs. These images were used to quantify centrilobular fibrosis and the sinusoidal area. The images were subsequently analyzed using the macro-batch mode of ImageJ^®^ (version 1.53e, © National Institutes of Health, Bethesda, MD, USA). 

#### 4.5.3. Histological Evaluation

MCT-induced pulmonary arterial hypertension and right ventricular remodeling was previously reproduced by our research group using the same protocol as described here. The evaluation of the liver and heart histological slides was performed by two experienced pathologists who had no access to the clinical data or the experiment results. 

To quantify cardiac wall thickness and dilatation, the right ventricular free wall thickness (RVFWT) and chamber area (RVCA) were measured at cross-section at the middle third of the ventricles using ZEN 2.3 (blue edition software). The RVFWT was measured at the central region of the RV free wall at 10× magnification and expressed in micrometers (µm). The RVCA was also measured at 10× magnification and expressed in square micrometers (µm^2^).

An accurate analysis of congestive hepatopathy was performed by reviewing common histological liver features (qualitative and semiquantitative parameters) and evaluating hepatic fibrosis, sinusoidal dilatation and hepatocyte count through a morphometric analysis. 

Two methods were used to assess t hepatic fibrosis: Congestive Hepatic Fibrosis Score (CHFS), described by D-F Dai et al. [6]; and Centrilobular Fibrosis Ratio (CFR), described in the sequence. The CHFS is a semi-quantitative pathological classification method. Based on the pattern of fibrosis, scores of 0, 1, 2, 3, and 4 may be assigned as follows: score 0, no fibrosis; score 1, central zone fibrosis; score 2, centrilobular and portal fibrosis; score 3, bridging fibrosis; score 4, cirrhosis.

The following histopathological parameters were reviewed: subendothelial hemorrhage of central venule (CV), sinusoidal dilatation, sinusoidal congestion, centrilobular hemorrhagic necrosis and steatosis. Subendothelial hemorrhage: 0, absent; 1, mild (minority of CV involved); 2, moderate-severe (majority of CV involved). Sinusoidal dilatation and congestion: 0, absent; 1, mild (centrilobular involvement limited to one-third of lobular surface); 2, moderate (centrilobular involvement extending in two-thirds of the lobular surface); 3, severe (complete lobular involvement). Centrilobular hemorrhagic necrosis: 0, absent; 1, mild (minority of centrilobular zones involved); 2, moderate-severe (majority of centrilobular zones involved). Steatosis: 0, absent (<5%); 1, mild (5–33%); 2, moderate (>33–66%); 3, severe (>66%) [26,35,36,37]. The final histological scores of each group were obtained using the mode of the scores of the 10 liver slides (20 cross-sectional liver lobes).

##### Quantification of Sinusoidal Lumen Ratio (SLR) and Centrilobular Fibrosis Ratio (CFR)

All the images were analyzed using an in-house-developed macro, written to quantify absolute value in micrometers and the percentage of centrilobular fibrosis area and sinusoidal lumen area compared to the total amount of tissue area within a ROI, allowing the measurement of the CFR and SLR in each visual field. The means of five visual fields were compared.

For all the images analyzed, the macro first set a scale for the image (4.55 µm/pixel). Next, the macro applied a specific colour threshold algorithm. After obtaining all the individual components and correcting the images of the areas to be excluded, the macro assessed the areas of the all components in µm^2^ and percentages. The threshold used by the macro was set empirically by analyzing a test set of 50 images and selecting the threshold which identified the components most effectively. 

**Figure 7:**
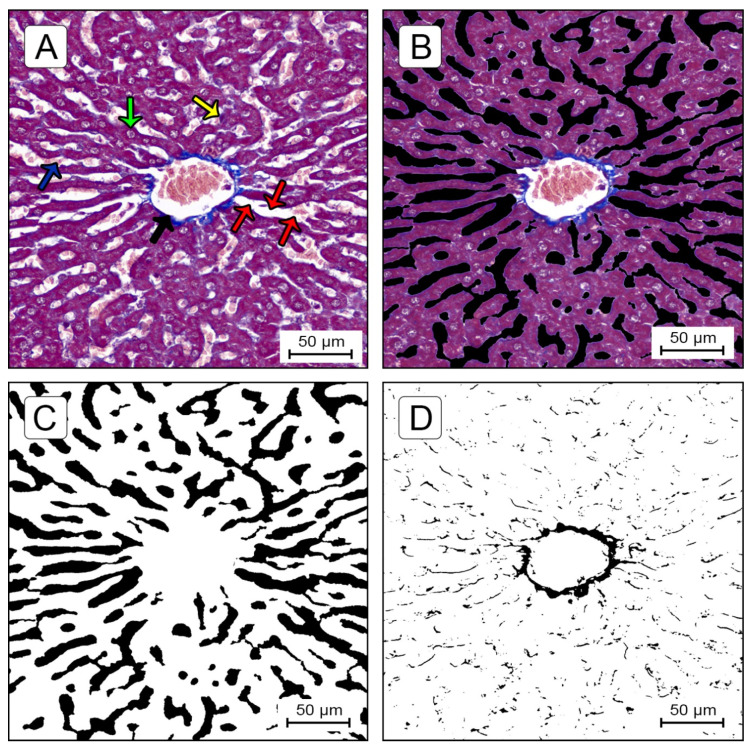
(**A**). TM-stained specimen of liver tissue; centrilobular vein (black arrow); sinusoids (red arrows); hepatocyte (green arrow); Kupffer cell (yellow arrow); red blood cells in sinusoidal lumen (blue arrow). (**B**) Manually extracted sinusoidal lumen image using Adobe^®^ Photoshop^®^. (**C**) Sinusoidal mask image using ImageJ^®^; black pixels indicate the sinusoidal lumen and white pixels indicate the trabecular structures and central vein. (**D**) Fibrotic zone mask image using ImageJ^®^; the black pixels indicate fibrotic zones.

An MT-stained image of liver tissue specimen is shown in Figure 7A. The connective tissue is stained blue, hepatocyte nuclei (also lymphocytes and Kupffer cells) are stained dark red/purple and cytoplasm is stained red/pink/purple. Sinusoids and fat droplets generally appear as white areas. A trabecula was considered as a series of cells segmented by sinusoids and stromata. To quantitatively measure a sinusoid lumen area, it is necessary to segment sinusoids from the trabecula structure. However, as cytoplasm texture depends on the conditions of the cells (i.e., steatosis, atrophy, fatty metamorphosis, dysplasia) and specimens (staining time or specimen fixation), accurate automatic extraction can be a difficult task. Therefore, for the measurement of the sinusoidal area, the content of the sinusoidal space in the selected images (red blood cells, lymphocytes, artifacts and others) were manually extracted and filled with black pixels (Figure 7B), using Adobe^®^ Photoshop^®^ CS6 (version 13.0, © Adobe Systems Incorporated, 1990–2012, San Jose, CA, USA). In Figure 7C, the black pixels indicate the sinusoidal lumen and the white pixels indicate the trabecular structures and central vein. The colour threshold application settings in ImageJ^®^ software for measuring the sinusoidal lumen area were: hue 0–255, saturation 0–255, brightness 0–50 (Figure 7C). In Figure 7D, black pixels indicate the fibrotic zones. The application settings for measuring fibrosis were: hue 140–190, saturation 0–255, brightness 0–248.

##### Quantification of Hepatocyte Nuclei

To assess the number of hepatocytes per field and, consequently, cell death, the images of the HE-stained liver sections were examined by using ZEN 2.3 (blue edition) software (© Carl Zeiss Microscopy GmbH, 2011) and a manual count was performed. The cell numbers were expressed as Hepatocyte Cell Ratio (number of hepatocytes per field analyzed). Apoptotic cells and hepatocytes undergoing necrosis were not counted. Apoptotic cells were identified by using morphological criteria, such as cell shrinkage, chromatin condensation and margination and apoptotic bodies, while hepatocytes undergoing necrosis were identified by using the following criteria: increased eosinophilia, cell swelling and lysis, loss of architecture, karyolysis and karyorrhexis [38].

### 4.6. Statistical Analysis

The continuous variables were given as mean ± standard deviation (SD). The continuous parameters were summarized by randomization using descriptive statistics. The Kolmogorov–Smirnov test was used to assess whether the variables were normally distributed. The Kruskal–Wallis method, followed by the Simes–Hochberg method or ANOVA one-way, followed by the Tukey test, were used for intergroup comparison, according to whether the data were normally distributed. The survival rate was presented as a Kaplan–Meier curve. The correlations were evaluated through linear regression analysis. The data were analyzed using Pearson’s Correlation Coefficient (r) and Coefficient of Determination (R^2^). Probability values of less than 0.05 were considered significant. All the statistical analyses were carried out using Action Stat^®^ software (version 3.7, Estatcamp Team (2014), Software Action, Estatcamp–Statistics and Quality Consultancy, São Carlos–SP, Brazil) and the graphics were created using MedCalc^®^ (version 19.3.1, © MedCalc Software Ltd., 1993–2020, Ostend, Belgium).

The sample size and allocation into groups were conducted based on the experience of our research group in similar studies involving animal models, specifically the monocrotaline-induced PAH model9, on the statistical viability of the experimental design, on previous medical research and on the norms established by the NIH guidelines and CEUA/FEMPAR.

## 5. Conclusions

In summary, right ventricular hypertrophy related to monocrotaline-induced pulmonary arterial hypertension caused congestive hepatopathy in a rat model. The model successfully reproduced several histological and structural changes in liver parenchyma similar to those found in patients with right heart disfunction, such as sinusoidal dilatation and congestion, centrilobular fibrosis and hepatocyte atrophy. Furthermore, this study found a moderate-strong positive correlation between SLR and CFR and morphometric right ventricle parameters.

## Figures and Tables

**Table 1:** Semiquantitative evaluation of histological parameters for the assessment of congestive hepatopathy.

Histological Parameters	Groups					*p*-Value
	CG	MCT_15_	MCT_30_	MCT_37_	MCT_ED_	
CHFS	−	−	+	+	++	*p* < 0.05 ^a–d^
	0 ± 0	0.2 ± 0.42	0.6 ± 0.52 ^a,b^	1.1 ± 0.57 ^a–c^	1.9 ± 0.32 ^a–d^	
SH	−	−	+	++	++	*p* < 0.05 ^a–d^
	0 ± 0	0.1 ± 0.32	0.8 ± 0.79 ^a,b^	1.3 ± 0.95 ^a,b^	1.8 ± 0.63 ^a–c^	
SC	−	+	++	+++	+++	*p* < 0.05 ^a–d^
	0.1 ± 0.32	1.1 ± 1.10 ^a^	1.5 ± 0.97 ^a^	2.3 ± 0.67 ^a,b^	2.9 ± 0.32 ^a–c^	
SD	−	−	+	++	+++	*p* < 0.05 ^a–d^
	0.1 ± 0.32	1.4 ± 0.52 ^a^	1.9 ± 0.57 ^a,b^	2.5 ± 0.71 ^a–c^	3.0 ± 0.00 ^a–d^	
CHN	−	−	−	+/++	++	*p* < 0.005 ^a–d^
	0 ± 0	0 ± 0	0 ± 0	0.7 ± 0.82 ^a-c^	1.7 ± 0.67 ^a–d^	
Steatosis	−	−	+	+	++	*p* < 0.05 ^a–d^
	0 ± 0	0 ± 0	1.6 ± 0.63 ^a,b^	1.5 ± 0.53	1.5 ± 1.01 ^a,b,d^	

Note: Parameters were given a score of 0 (−) for absent, 1 (+) for mild, 2 (++) for moderate, or 3 (+++) for severe changes (except for SH and CHN, which were awarded a different score), as described in the Materials and Methods section. Values are presented as the mean ± SD and mode. CHFS, congestive hepatic fibrosis score; CHN, centrilobular hemorrhagic necrosis; SC, sinusoidal congestion; SD, sinusoidal dilatation; SH, subendothelial hemorrhage. ^a^ *p*-value was compared to the CG. ^b^ *p*-value was compared to the MCT_15_ group. ^c^ *p*-value was compared to the MCT_30_ group. ^d^ *p*-value was compared to MCT_37_ group (all the *p*-values were obtained using the Kruskal–Wallis method followed by the Simes–Hochberg method for intergroup comparison).

## Data Availability

All the data generated or analyzed during this study are included in this published article.
